# T135. NEIGHBORHOOD-LEVEL PREDICTORS OF AGE AT ONSET AND DURATION OF UNTREATED PSYCHOSIS IN FIRST-EPISODE PSYCHOSIS

**DOI:** 10.1093/schbul/sby016.411

**Published:** 2018-04-01

**Authors:** Benson Ku, Luca Pauselli, Marc Manseau, Michael Compton

**Affiliations:** 1 Hofstra Northwell School of Medicine; 2 Columbia University, New York State Psychiatric Institute; 3 New York State Office of Mental Health; 4 Columbia University

## Abstract

**Background:**

Recently, there has been increasing interest in the role of the social environment in the development and outcomes of schizophrenia. We investigated whether or not several neighborhood characteristics would be associated with two important prognostic factors in early-course psychosis, age at onset of psychosis (AOP) and duration of untreated psychosis (DUP). It is known that certain risk factors such as gender, family history, and history of cannabis use are associated with an earlier AOP and other risk factors such as history of being incarcerated, history of cannabis use, and mode of onset of psychosis (MOO) are associated with longer DUP. We sought to determine whether neighborhood characteristics would also be risk factors for these two prognostic indicators.

**Methods:**

Data were collected as part of the Atlanta Cohort on the Early Course of Schizophrenia project, which included patients hospitalized for a first episode of a schizophrenia-spectrum disorder. Diverse variables were obtained from interview-based measures, data from chart reviews, and informant/family member collateral interviews, including gender, family history of psychosis, history of cannabis use, history of incarceration, MOO, patient-level residential mobility, Neighborhood Disorder Scale (NDS), AOP, and DUP. We retrieved 13 neighborhood characteristics using census tract-level data from the American Community Survey linked to individual addresses. Factor analysis with orthogonal rotation produced four neighborhood-level factors. With the addition of NDS, five independent variables were entered into two separate linear regression models with AOP and DUP as the dependent variables respectively; final models were derived from stepwise backward elimination, controlling for known predictors of AOP and DUP, and individual-level socioeconomic variables.

**Results:**

Reliable census tract data were available for 143 participants. For the linear regression model pertaining to AOP, after stepwise backward elimination, the remaining independent predictor was neighborhood-level residential instability (β=-0.210; p=0.018). This variable remained significant after controlling for known risk factors such as gender, family history, and age at first cannabis use (β=-0.237; p=0.017) and after controlling for patient-level residential mobility (β=-0.195; p=0.031). Regarding the linear regression model for DUP, after stepwise backward elimination, the remaining independent predictors were the General Socioeconomic Status neighborhood factor (β=0.269; p=0.007), the Low Household Value neighborhood factor (β=0.190; p=0.046), and NDS (β=0.339; p<0.001). After controlling for known predictors of DUP, including MOO, history of incarceration, and age at first cannabis use, NDS and MOO remained significant (NDS: β=0.326; p=0.008; MOO: β=0.466; p<0.001).

**Discussion:**

We found initial evidence that neighborhood-level characteristics are associated with important outcomes that affect the prognosis of early psychosis. Residential instability was associated with an earlier AOP and perceived neighborhood disorder was associated with a longer DUP. The association between the social environment and prognostic factors, widely explored in other health conditions, may have significant implications on the understanding and management of psychosis and should be further explored. Area-level context, beyond the individual level, might be considered when implementing services and policies that could contribute to improved outcomes among those with early schizophrenia.

